# Diet Quality among Women with Previous Gestational Diabetes Mellitus in Rural Areas of Hunan Province

**DOI:** 10.3390/ijerph17165942

**Published:** 2020-08-16

**Authors:** Mingshu Li, Jingcheng Shi, Jing Luo, Qing Long, Qiping Yang, Yufeng OuYang, Hanmei Liu, Qian Lin, Jia Guo

**Affiliations:** 1 Department of Nutrition Science and Food Hygiene, Xiangya School of Public Health, Central South University, 110 Xiangya Road, Changsha 410078, Hunan, China; limingshu@csu.edu.cn (M.L.); luojing2546@csu.edu.cn (J.L.); yangqiping12@csu.edu.cn (Q.Y.); oyyf0102@csu.edu.cn (Y.O.); hanmeiliu@csu.edu.cn (H.L.); 2 Department of Epidemiology and Health Statistics, Xiangya School of Public Health, Central South University, 110 Xiangya Road, Changsha 410078, Hunan, China; shijch@csu.edu.cn; 3 Xiangya School of Nursing, Central South University, 172 Tongzi Road, Changsha 410013, Hunan, China; longqing226@163.com

**Keywords:** diet quality, Chinese Healthy Eating Index (CHEI), women with previous gestational diabetes mellitus (GDM), Hunan, rural areas

## Abstract

Diet quality is critical for postpartum type 2 diabetes mellitus (T2DM) progression among women with a history of gestational diabetes mellitus (GDM). The Chinese Healthy Eating Index (CHEI) is a dietary index developed according to the latest Chinese Dietary Guidelines (CDG-2016). Our study aimed to assess the diet quality of women with previous GDM in rural areas of Hunan Province by applying the CHEI. Women with previous GDM in two counties of Hunan were enrolled. Their food intake data, which were used to calculate their CHEI scores, were collected by a 24-h dietary recall. The association of CHEI with sociodemographic and anthropometric variables was evaluated via linear regression models. 404 women were included in the final analysis. The mean score of the total CHEI was 54.9 (SD 7.9). The components of whole grains and mixed beans, seeds and nuts, tubers, dairy, and poultry scored extremely low. Ethnic minority groups and women younger than 30 years had lower CHEI scores. Our study observed an unsatisfactory diet quality among women with previous GDM in rural areas of Hunan Province. Future dietary education focusing on the CDG is needed to improve their diet quality and help in T2DM prevention among this population, especially young and ethnic minority women.

## 1. Introduction

Women with previous gestational diabetes mellitus (GDM) have a sevenfold higher risk of type 2 diabetes mellitus (T2DM) progression [1,2]. To postpone/prevent postpartum T2DM, they should follow healthful dietary behaviors [3,4]. The 10-year Diabetes Prevention Program demonstrated that intensive lifestyle intervention (diet and physical activity) reduces progression to T2DM by 35% among women with a history of GDM [5]. There is no universal dietary pattern recommended for this population; nonetheless, based on guidelines and major randomized controlled trials (RCTs), the key elements of a proper diet include limiting calories, fats, alcohol, monosaccharides, and red meat and increasing vegetables, whole grains, soybeans, dairy, and nuts [6,7,8,9].

Diet indices are comprehensive tools used to quantify the diet quality and predict the health outcomes of women with previous GDM [10,11]. In the cohort of Nurses‘ Health Study II, Deirdre, K. et al. applied three indices—namely, the Alternate Healthy Eating Index (AHEI), Dietary Approaches to Stop Hypertension (DASH), and the Alternate Mediterranean Diet (AMED) [11]—and revealed that all three indices are inversely associated with postpartum T2DM progression. Among these indices, AHEI has the strongest association with T2DM, and participants with the highest level of AHEI (quartile 4) showed a 57% lower risk of T2DM compared with those with the lowest AHEI level (quartile 1). Although they are well established and extensively applied, these indices may not be suitable for direct usage among the Chinese because their food components and quantity were designed for Western individuals.

The Chinese Healthy Eating Index (CHEI) was developed in 2017 [12] (Table 1) by referring to the methodology of HEI and the recommendation of the Chinese Dietary Guidelines (CDG-2016) [13]. For the Chinese population, CDG-2016 recommends the Balanced Dietary Pattern as an ideal dietary model. Specifically, Chinese adults should eat at least 12 different foods on a daily basis. Increasing the intake of whole grains, vegetables, fruits, and fish and seafood is suggested. Salt, cooking oils, refined grains, and red meat should be consumed with limitation. CHEI measures one’s compliance with the Balanced Dietary Pattern by scoring 17 components (12 adequacy components and five moderation components). Many of the components are consistent with those of international indices. For example, CHEI and AHEI-2010 have seven common components, namely, alcohol, red meat, sodium, nuts and legumes, fruits, vegetables, and whole grains (Table A1). CHEI has been proposed to be a proper tool to measure the diet quality of the general and special Chinese populations. The developer of CHEI applied this index to participants of the China Health and Nutrition Survey (CHNS-2011). Most participants scored between 40 and 60 (mean value 52.4) and showed a highly insufficient intake of whole grains and mixed beans, fruits, dairy and poultry and excessive consumption of red meat, sodium and cooking oils [12]. This dietary index has also been employed among rural residents in Xinjiang (northwest China), with a mean value of 47.9 [14].

China has witnessed a tremendous increase in the prevalence of T2DM—from 0.67% in 1980 to 10.4% in 2013 [4]. For rural regions, the issue is even more urgent, since traditional lifestyles are changing in parallel to fast urbanization, and the growth of T2DM incidence in rural areas has outpaced that in urban areas [4,15]. Nearly one-fifth of pregnant women are diagnosed with GDM in China [16,17], and they are a large high-T2DM-risk population. However, no study has examined the status of their lifestyles. In this study, we aimed to evaluate the diet quality of women with previous GDM in two counties of Hunan Province by applying CHEI and identify influential factors.

## 2. Materials and Methods

### 2.1. Study Design and Ethics Approval

This study is a baseline survey on the dietary practice of women with a history of GDM. This work is part of a randomized clinical trial (ChiCTR1800015023) that aims to examine the effect of intensive lifestyle modifications on physiological health outcomes (e.g., T2DM development, insulin resistance, and weight-related variables) for women with previous GDM in rural areas [18]. According to a previous study that applied CHEI to the 2011 China Health and Nutrition Survey, which obtained standard deviations (SDs) of 9.5 among rural residents and 11.3 among female participants [12], we calculated a sample size of 423 under a hypothesized SD of 10.0, allowance error of 1.0, and drop-out rate of 10% (PASS software version 11.0 for Windows, NCSS LLC, Kaysville, UT, USA). Ethics approval was obtained from ethical committee of Xiangya Nursing School of Central South University (No. 2016034).

### 2.2. Participants and Recruitment

We chose two study sites located in the eastern and western areas of Hunan Province to represent the different socioeconomic status, lifestyles and ethnic groups in rural regions of Hunan. They are General Hospital of Youxian County and Maternal and Children’s Hospital of Yongding County. Adult women who were previously diagnosed with GDM through their medical records in these sites were introduced to our study by trained nurses. They decided whether to join our study by personal discretion, and those who agreed to participate were required to sign an informed consent form. Women who met any of the following criteria were excluded: diagnosed with T2DM before pregnancy or after delivery, pregnant or lactating, had physical or cognitive disability, or used concomitant drugs that influenced glucose metabolism or weight.

### 2.3. Data Collection

#### 2.3.1. Questionnaires Survey

All participants were instructed to fill out self-report questionnaires asking for sociodemographic information (e.g., age, ethnicity, education, occupation, civil status, family income, and number of children) and medical history (e.g., measures to control GDM, concomitant diseases, and medication history).

#### 2.3.2. Anthropometric Measurements

Anthropometric parameters (body weight and height) were measured and recorded by trained investigators who were graduate students of public health and nurses. Body mass index (BMI) was calculated by dividing the weight (in kilograms) by height (in meters squared). According to Chinese guidelines, BMI ≥28 kg/m^2^ indicates obesity, and 24–27.9 shows overweightness [19].

### 2.4. Dietary Assessment 

The dietary intake of each participant was collected via a 24-h dietary recall administered by trained investigators. Before interviews, the participants were required to take pictures of all the foods (including beverages) consumed in three consecutive days (two workdays and one weekend day). During the interviews, the participants showed the pictures and recalled their food quantities to investigators who filled out forms of 24-h dietary recalls.

The nutrient content of the food was estimated using the NutriStar software (Shanghai Zhending Inc., Shanghai, China) based on the 2018 Chinese Food Composition Table, which contains 1506 unique Chinese foods [20].

### 2.5. Dietary Quality

The Chinese Healthy Eating Index (CHEI) was applied to evaluate dietary quality. Total CHEI score was the sum of scores of 17 components. Investigators assigned 0–5 or 0–10 points to the participants at each component based on their food intake (standard portion/day). For 12 adequacy components (total grains, whole grains and mixed beans, tubers, total vegetables, dark vegetables, fruits, dairy, soybeans, fish and seafood, seeds and nuts, poultry, and eggs), point 0 was assigned for no intake, the maximum point was given where the intake met the recommendation, and the other scores were allotted proportionately. As to the five moderation components (red meat, cooking oils, sodium, added sugars, and alcohol), the points were reversed (10–0 or 5–0). Women with complete adherence to the intake recommendation would receive 5 or 10 points. For those who consumed excessively, the more they consumed, the fewer point they received. The full score of CHEI is 100, indicating optimal diet quality that has great consistency with the dietary guidelines. 

### 2.6. Statistical Analysis

Continuous variables were described by mean (SD) or median (95% confidence interval). Categorical variables were summarized with counts and percentages. The Kolmogorov–Smirnov (K–S) test was used to assess whether the total CHEI score and component scores had a normal distribution. If a normal distribution was confirmed, the scores would be described by the mean; otherwise, they would be described by the median. The patients were categorized by terciles of the total CHEI score (high, intermediate and low). To compare the food and nutritional intake among these three groups, we applied one-way ANOVA or the Kruskal–Wallis (K–W) test. ANOVA would be used under circumstances with a homogeneous variance, and the K–W test would be used for heterogeneous variances. The energy and nutritional intake was categorized as insufficient, adequate and excessive by comparing it with CDG-2016’s recommendation, where insufficient intake was defined as less than 90% of EER/RNI or the lower cutoff of AMDR and excessive intake was defined as more than 110% of EER/RNI or the higher cutoff of AMDR. Multiple linear regression (stepwise method) was applied to analyze the association between the CHEI score and dietary nutrients intake. In this analysis, the dependent variable was the CHEI score, and the independent variables were energy, protein, fat, carbohydrate, dietary fiber, iron, zinc, copper, magnesium, selenium, calcium, sodium, phosphorus, iodine, vitamin A, thiamine, riboflavin, vitamin C, vitamin E, saturated fatty acid, monounsaturated fatty acid, polyunsaturated fatty acid, niacin, and cholesterol. The models were deemed significant if ANOVA sig. < 0.05. Among the significant models, we chose the one with the highest R^2^ value, which indicated model fitness. To identify the sociodemographic/anthropometric factors of CHEI, we applied linear regression models and included age, ethnicity, education level, occupation status, family income, measures to control GDM, and number of children as potential influential factors. Factors with no significant association with the CHEI score in univariate regression analysis were excluded, and the remaining factors were further included in a multivariate model. Statistical analysis was conducted via SPSS (version 24), *p* value < 0.05 was considered statistically significant.

## 3. Results

### 3.1. Characteristics of Study Population

A total of 461 women were enrolled in this study, and 57 were excluded from the final analysis due to the current diagnosis of T2DM or incomplete dietary information. The characteristics of the 404 remaining women are shown in Table 2. The mean age of the participants was 31.3 ± 5.1 years. Nearly half of the women were ethnic minorities (45.7%). Most of the women (77.2%) received education of senior high school or above (>9 years). One-third of the participants were full-time housewives, and the rest of them mainly worked in factories (19.4%) and fields (11.5%) or were self-employed (15.0%). Moreover, 27.3% of the families obtained a monthly income of less than 3000 yuan (equal to $420 USD) (the average disposable income of Chinese residents was 25,974 yuan/year in 2017) [21]. The mean BMI was 23.9 (SD 3.7), and the proportions of participants who were overweight or obesity were 32.4% and 13.8%, respectively.

### 3.2. Total CHEI and Components Scores

The total CHEI scores among the 404 participants had a normal distribution (K-S test, *p* = 0.200). None of the component scores had a normal distribution. The total CHEI scores ranged from 33.6 to 78.2, with a mean value of 54.9 (SD 7.9). The median value of the component scores is in Table 3. Overall, the scores were quite low across the adequate components, five of them scored zero at the median level (whole grains and mixed beans, tubers, dairy, seeds and nuts, poultry). The proportion of participants who received zero points was the highest for whole grains and mixed beans (89.9%), followed by dairy (75.0%), seeds and nuts (67.1%), poultry (65.6%), tubers (56.7%), fish and seafood (44.8%), and fruits (39.9%). In terms of the moderation components, the scores were generally high for alcohol and added sugars. 99.8% and 98.8% of the participants obtained full score at alcohol and added sugars, while only 12.6% and 49.0% at red meat and sodium.

### 3.3. CHEI Component Foods Intake 

The intake of 17 CHEI component food groups in the low-, intermediate-, and high-CHEI-scoring groups is demonstrated in Table 4. Numerically, participants with higher total CHEI scores consumed more tubers, total vegetables, dark vegetables, fruits, dairy, eggs, seeds and nuts, fish and seafood, and poultry, and they ate less red meat, added sugars, and sodium. The intake of these foods was statistically different among three groups except that of added sugars. Relative to CDG recommendations, most of the adequate components were consumed insufficiently. For example, the average intake of whole grains and mixed beans (1.9 g/day) and dairy (28.5 g/day) was less than one-tenth of the recommendation. The participants consumed 196.1 g and 68.5 g of total vegetables and fruits on a daily basis, while the CDG recommendation were 300–500 g and 200–350 g, respectively. 

### 3.4. Nutrition Intake and Its Association with Total CHEI Score

The energy and nutrient intake of the participants is shown in Table 5. In particular, 75.7% and 86.4% of the women consumed excessive fat and saturated fat. Meanwhile, for micronutrients, 42.7–96% of them took calcium, iron, vitamin A, thiamine, riboflavin and vitamin C from food inadequately. As in the case of foods intake, we observed a trend wherein participants with higher CHEI scores obtained more protein, dietary fiber, animal protein, monounsaturated fatty acids, polyunsaturated fatty acids, iron, calcium, vitamin A, thiamine, riboflavin, vitamin C, and vitamin E.

The association between the total CHEI score and dietary nutrients was examined by a multiple linear regression test that implied vitamin E, calcium, riboflavin, sodium, saturated fatty acid, and zinc associated with the CHEI score significantly (ANOVA sig. 0.000; R^2^ 0.484). The regression formula was as follows:

CHEI score = 49.361 * + 0.366 X_1_ + 0.020 X_2_ + 7.222 X_3_ − 0.006 X_4_ − 0.102 X_5_ − 0.480 X_6_ − 0.016 ** (1)

*, intercept; X_1_, intake of vitamin E; X_2_, intake of calcium; X_3_, intake of riboflavin; X_4_, intake of sodium; X_5_, intake of saturated fat; X_6_, intake of zinc; **, residue.

The intercept of regression formula was 49.361, the residue value was −0.016. The coefficient value of vitamin E, calcium, riboflavin, sodium, saturated fatty acid, and zinc were 0.366, 0.020, 7.222, −0.006, −0.102, and −0.480, respectively, indicating that vitamin E, calcium, and riboflavin contributed to the CHEI score positively, while sodium, saturated fatty acid, and zinc linked to the CHEI score negatively.

### 3.5. Association of Sociodemographic, Anthropometrics, and CHEI Score

The distribution of the CHEI level in different groups of educational background, occupation, family income, number of children, family history of T2DM, GDM control, and BMI level was similar. A larger proportion of participants who were older than 30 years or were of Han ethnicity displayed a higher level of CHEI than those among participants who were younger than 30 years or were from minority ethnicity groups (37.5% vs. 30.2%; 42.9 vs. 22.8%). The association of CHEI with age and ethnicity was significant in the multivariate model (*p* = 0.028, 0.000) (Table A2).

## 4. Discussion

As the first study assessing the dietary quality of Chinese women with previous GDM, our study revealed an alarming gap between the optimal and actual dietary quality. Generally, the intake of whole grains and mixed beans, tubers, dairy, fruits, and seeds and nuts was extremely low, whereas red meat and sodium were overconsumed. The average total score of CHEI was only 54.9. Although CHEI does not have cut-off points corresponding to good or poor diet quality, we may refer to the categorization of HEI, namely good, requiring improvement or poor (HEI, >80, 51–80, <50) [22]. Among the participants in our study, 67.5% required diet improvement and 32.5% had poor diet quality, none of their diet quality was good. Our results were consistent with previous findings in other countries [11,23]. In Nurse’ Health Study II, the mean AHEI-2010 at quartile 4 of 4413 women with a history of GDM was only 52.4 [11]. By reviewing 18 research articles, Kaiser et al. found that only 5–31% women take five food groups of fruits and vegetables postpartum [23]. Our study did not explore the barriers to a healthful diet, but we expected that there are some common issues for women with a GDM history across countries, such as financial constraints, childcare duties, and dietary preferences of other family members [23,24].

Chinese diet is characterized by a cereal-based pattern. White rice, commonly made of refined grain, is the daily food for most of the participants (98.0%) in our study. The average intake of white rice was 325.5 g/day; 53.6% (*n* = 257) of the participants ate more than 420 g of white rice every day. Excessive consumption of white rice is related to an increased risk of T2DM. Nanri and Villegas observed a significant increase in T2DM risk in women who consumed more than 300 g/day or 420 g/day of white rice [25,26]. White rice is primarily composed of starchy endosperm, with bran and germ removed during the refining process. The physical and botanical structures of cereal have been proven to affect insulin metabolism [27]. A previous study demonstrated that glucose-dependent insulinotropic polypeptide (GIP) and glucagon-like-peptide 1 (GLP-1), both potent hormones regulating postprandial insulin release, responded at lower levels after consumption of white wheat bread compared with whole-kernel/whole-meal rye bread [28]. Moreover, the loss of ingredients such as cereal fiber, magnesium, and lignan leads to impaired insulin sensitivity and faster gastric emptying [29,30]. Although the intake of total grains was generally high among the participants, the whole grains consumption (mean: 1.8 g/day) was far from the recommended level of 48–80 g [31], and even lower than the average level in the general Chinese population (4.6 g/day) [32]. The explanation might be that people who habitually eat white rice would have decreased access to other beneficial cereals and nutrients, such as whole grains. Moreover, the whole-grain food industry is still in the early stage of development in China, with many challenges in terms of industrial standard, processing and storage [33,34]. For people living in rural regions, whole-grain food might be less accessible for promotional and economic reasons.

Besides whole grains, component of dairy had extremely low scores. Dairy is a major source of calcium, which improves the pancreatic beta cell function and peripheral insulin sensitivity [35]. Although the relationship of serum calcium and T2DM has not been concluded, the 10-year Ansung–Ansan cohort confirmed that higher dietary calcium intake leads to decreased T2DM risk [36]. Other nutrients contained in dairy, such as whey proteins, vitamin D and magnesium contribute to insulin secretion and glucose control synergistically or independently [37,38,39]. Several meta-analyses confirmed the association of dairy intake and T2DM [40,41]. Schwingshackl found dairy intake up to 400–600 g/day would decrease the risk of T2DM by 6% [41]. The recommended level in Chinese guideline is slightly lower, 300 g/day, but 99% (400/404) of the participants failed to meet it. This phenomenon was mainly attributable to the limited supply. Although nearly one-fifth of the world population are Chinese, the dairy production in China had been accounting for only around 3.5% of world dairy production for a long period [42]. In early 2000s, the Chinese government initiated the “A glass of milk, a stronger nation” campaign. Since then, there has been an increase in the dairy intake across the nation [43]. By comparing our findings with those of the 2010–2012 CHNS, we also saw this trend in the rural residents (12.1 g/day vs. 28.5 g/day).

Less than one-fifth of the participants met the CDG’s recommendation for vegetables and fruits, reflecting a very low consumption of these foods. Only 20.7% (*n* = 84) of them reported eating at least five kinds of fruits or vegetables, similar to the results of Kaiser, B. [23]. A higher intake of vegetables, especially green leafy vegetables, helps lower T2DM progression [44,45,46]. Although the relationship of fruits and T2DM is inconsistent among studies [47,48,49], systematic reviews suggest that increasing the intakes of fruits up to 200–300 g/day lowers the risk of T2DM by 10% [41,44,45]. Evidence shows the carotenoid and vitamin C in vegetables and fruits are critical factors for T2DM prevention by resisting oxidative stress, reducing systemic inflammation, and modulating the toxicity of polychlorinated biphenyls [50,51,52]. Recently, a case-cohort study from eight European countries (*n* = 23,416) revealed that higher level of total carotenoids and vitamin C in plasma are associated with 25% and 18% lower risk of T2DM, respectively [53]. For many Chinese people, especially those living in rural regions, fresh vegetables and fruits are not a necessity for daily meals, mainly because they do not fully understand the beneficial effect to health.

Several nutrients, namely vitamin E, calcium, riboflavin, sodium, saturated fatty acid, and zinc, contributed to the CHEI score significantly. Most of these nutrients were consumed improperly. The issue of fat consumption was concerning. In our study, the fat %TE echoed the results in other surveys demonstrating “high-fat” as a character of Chinese dietary patterns in recent years [15,54]. Moreover, suboptimal quality was conspicuous, given that the average SFA%TE was twice the CDG recommendation, and the ratio of SFA: MUFA: PUFA was 2:1:2. The impact of SFA on insulin sensitivity or secretion is yet to be determined [55,56]. The improvement of insulin sensitivity for dietary MUFA has been well established and is partly attributed to conserving the insulin receptor substrate-1/phosllatidylinostitol-3-kinase insulin signaling pathway and mitigating β-cell hyperactivity [57,58,59]. SAF should be limited to under 8–10% of total energy, and it should be replaced with MUFA as SFA was progressively decreased in the diet [3]. Vegetable oils, fish and seafood, and nuts are rich in MUFA and PUFA. Many of the participants in our study consumed animal oils instead of vegetable oils, and the daily consumption of nuts and fish and seafood was generally below the recommended levels. Over 80% of the participants absorbed calcium and riboflavin insufficiently from their foods, which might be explained by their low intake of dairy, eggs, organ meats, and dark vegetables. Zinc deficiency contributes to a higher risk of T2DM [60,61]. In our study only 6.5% of the participants had inadequate zinc, but 88.1% of them had excessive zinc. This phenomenon might be associated with their high consumption of red meat.

Nearly half of the participants were from minority ethnic groups, and these women presented poorer diet quality than the Han participants. Previous studies show that children in rural and ethnic minority areas in central and western China have lower dietary diversity scores than those of their peers in other areas [62,63]. Investigators from the United States and Europe have also found that ethnic minority groups have poorer diet quality than those of ethnic majority groups [64,65,66]. Recent studies reveal ethnicity as an independent factor for diet quality [67,68]. In our study, all of the women from ethnic minorities were from Yongding County. This county is located in Tujia-Miao Autonomous Prefecture, where Tujia and Miao minorities account for 78.4% of the total population [69]. Traditional Tujia and Miao diet are characterized by cereals and smoked and salted foods [70]. Rice or glutinous rice are their staple food. Smoked meat and pickle kimchi are kept as an important part of the traditional diet culture. Food groups have been limited mainly because of geographic location and poor transportation. Age was another sociodemographic factor for the CHEI score. It is worth mentioning that, our study did not identify other socioeconomic elements (e.g., family income, educational level) as influential factors for dietary quality [71,72]. This finding is not fully consistent with previous reports, and we assumed the main reason was the relatively small sample size after stratification in our study.

This work has several limitations. We collected information relevant to food intake by 24-h dietary recalls. To aid in the food recall, we introduced visual reminders and study nurses, but we could not eliminate memory bias or misreporting. Moreover, this study was conducted in only one province, the participants were geographically concentrated and homogeneous. Thus, the result of our study might not be generalizable to other regions. Finally, although there is emerging evidence of an association between CHEI and metabolic syndrome [14,73], we cannot predict the exact T2DM progression risk for our study participants as users of other well-recognized measures [74]. We will follow-up with our participants for one and half years to gain more insight into its relationship with health outcomes as the trial proceeds.

## 5. Conclusions

We observed an unsatisfactory diet quality among women with previous GDM in rural areas of Hunan Province, as manifested by extreme deficiencies in whole grains and mixed beans, seeds and nuts, tubers, dairy, and poultry, as well as an overconsumption of red meat and sodium. Nutritional intake was generally inappropriate. Ethnic minority groups and women younger than 30 years were more likely to obtain lower CHEI scores. To improve the diet quality and prevent T2DM among women with previous GDM, further dietary education should focus on the latest Chinese dietary guidelines. In rural areas, nutritional knowledge training for primary health workers is crucial, since they are the principal executors of health interventions. Mobile-health interventions, such as Applications providing online dietician consultations and personalized menus, can be integrated into educational schemes to improve health care delivery.

## Figures and Tables

**Table 1:** Components of the Chinese Healthy Eating Index (CHEI) and scoring method [12].

Component	Maximum Points	Standard for Maximum Point	Standard for Zero Point
Adequacy			
Total grains	5	≥2.5 SP/1000 kcal	No intake
Whole Grains and mixed beans	5	≥0.6 SP/1000 kcal	No intake
Tubers	5	≥0.3 SP/1000 kcal	No intake
Total vegetables	5	≥1.9 SP/1000 kcal	No intake
Dark vegetables	5	≥0.9 SP/1000 kcal	No intake
Fruits	10	≥1.1 SP/1000 kcal	No intake
Dairy	5	≥0.5 SP/1000 kcal	No intake
Soybeans	5	≥0.4 SP/1000 kcal	No intake
Fish and Seafood	5	≥0.6 SP/1000 kcal	No intake
Poultry	5	≥0.3 SP/1000 kcal	No intake
Eggs	5	≥0.5 SP/1000 kcal	No intake
Seeds and Nuts	5	≥0.4 SP/1000 kcal	No intake
Moderation			
Added sugars	5	≤10% of energy	≥20% of energy
Sodium	10	≤1000 mg/1000 kcal	≥3608 mg/1000 kcal
Cooking oils	10	≤15.6 g/1000 kcal	≥32.6 g/1000 kcal
Red meat	5	≤0.4 SP/1000 kcal	≥3.5 SP/1000 kcal
Alcohol	5	≤15 g	≥40 g

**Table 2:** Demographic characteristics of the study population.

Variables	Total (N = 404) Mean (SD) or %
Age (years)	31.3 (5.1)
Ethnicity (%)	
Han ethnicity	54.3
Other ethnicities	45.7
Education (%)	
Junior high school or primary school (≤9 years)	22.8
Senior high school or junior college (9–12 years)	57.4
University (≥12 years)	19.8
Occupation (%)	
Unemployed	34.1
Employed	65.9
Marriage status (%)	
Married	99.3
Divorced	0.7
Monthly family income ($) (%)	
≤420	27.3
>420	72.7
BMI (%)	
<24	53.8
24–27.9	32.4
≥28	13.8
Age at GDM diagnosis (years)	30.3 (4.9)
Controlled GDM by diet regulation (%)	
Yes	62.8
No	37.2
Children Number (%)	
1	37.6
≥2	62.4

**Table 3:** CHEI components score among observed women.

Food Groups	Median	95% CI
Total grains	4.7	4.5, 4.9
Whole grains and mixed beans	0.0	0.0, 0.0
Tubers	0.0	0.0, 0.0
Total vegetables	2.2	2.1, 2.4
Dark vegetables	1.7	1.5, 1.9
Fruits	1.6	0.9, 2.1
Eggs	2.1	1.7, 2.3
Soybeans	1.5	1.3, 2.0
Dairy	0.0	0.0, 0.0
Seeds and nuts	0.0	0.0, 0.0
Fish and seafood	0.9	0.2, 1.4
Poultry	0.0	0.0, 0.0
Red meat	3.9	3.8, 4.1
Added sugars	5.0	5.0, 5.0
Cooking oils	10.0	10.0, 10.0
Alcohol	5.0	5.0, 5.0
Sodium	9.9	9.5, 10.0
Total CHEI	54.9 *	7.9+

* mean value. + SD value.

**Table 4:** Mean value of foods intake among low, intermediate, and high CHEI scoring groups.

Food Groups	Low CHEI Mean (SD)	Intermediate CHEI Mean (SD)	High CHEI Mean (SD)	CDG Recommendation (RNI/EER, AMDR) [13]
Total grains (g/d) *	228.9 (93.1)	259.1 (90.3)	228.5 (86.4)	250–400
Whole grains and mixed beans (g/d)	1.1 (4.9)	2.8 (9.5)	1.877 (6.5)	50–150
Tubers (g/d) *	9.6 (22.0)	18.8 (32.7)	34.0 (45.6)	50–150
Total vegetables (g/d) *	175.9 (127.8)	188.3 (119.4)	225.0 (128.3)	300–500
Dark vegetables (g/d) *	59.5 (57.7)	78.9 (80.9)	98.2 (8.5)	150–250
Fruits (g/d) *	32.8 (50.5)	57.2 (80.9)	115.4 (130.3)	200–350
Dairy (g/d) *	10.9 (38.2)	16.7 (46.7)	57.8 (99.9)	300
Soybeans (g/d) *	5.9 (8.8)	9.5 (12.9)	11.4 (13.8)	15–25
Eggs (g/d) *	21.2 (30.7)	28.3 (37.3)	33.3 (35.6)	40–50
Seeds and nuts (g/d) *	4.5 (10.9)	7.1 (16.3)	9.6 (16.1)	10
Fish and seafood (g/d) *	12.8 (29.9)	22.3 (29.9)	56.2 (150.6)	40–75
Poultry (g/d) *	8.4 (23.9)	18.1 (34.6)	24.9 (41.1)	40–75
Red meat (g/d) *	119.9 (84.1)	106.6 (66.6)	90.4 (61.8)	
Added sugars (g/d)	12.9 (1.1)	6.1 (5.2)	4.5 (7.4)	<=25
Cooking oils (g/d)	26.9 (7.1)	27.5 (8.6)	27.4 (6.3)	25–30
Alcohol (g/d)	1.4 (1.1)	0.3 (0.4)	0.1 (0.6)	<15 g
Sodium (mg/d) *	3081.3 (2216.6)	2503.0 (2186.1)	2304.6 (1373.2)	1500

* *p* < 0.05 for ANOVA or Kruskal–Wallis (K–W) test.

**Table 5:** Calorie and nutrient intake.

Dietary Parameter	Mean (SD)/ Median (IQR)	Prevalence (%)			CDG Recommendation (RNI/EER, AMDR) [13]
		Insufficient	Adequate	Excessive	
Energy (kcal)	1997.2 (727.0)	36.0	30.3	33.7	1800/2100/2400
Carbohydrate (%E)	51.8 (8.9)	39.0	54.1	6.9	50~65
Protein (g)	63.5 (29.4)	25.6	16.9	57.6	55
Fat (%E)	35.8 (8.2)	2.2	22.1	75.7	20~30
Saturated fat (%E)	16.2 (12.0)		13.6	86.4	<8
Calcium (mg)	346.7 (207.1)	96.0	2.0	2.0	800
Iron (mg)	19.2 (8.9)	42.7	22.8	34.5	20
Zinc (mg)	12.2 (5.0)	6.5	5.5	88.1	7.5
Vitamin A (mg)	394.8 (416.8)	73.7	9.9	16.4	700
Vitamin E (mg)	23.5 (7.9)	1.5	4.7	93.8	14
Thiamine (mg)	0.70 (0.4)	87.8	7.9	4.2	1.2
Riboflavin (mg)	0.70 (0.3)	83.9	10.9	5.2	1.2
Niacin (mg)	18.8 (9.0)	6.9	10.4	82.6	12
Vitamin C (mg)	73.8 (63.6)	61.5	13.2	25.3	100

## Appendix A

**Table A1:** Components and scoring mechanism of the Chinese Healthy Eating Index (CHEI) and the Alternate Healthy Eating Index (AHEI).

CHEI-2017				AHEI-2010			
Component	Maximum Points	Standard for Maximum Point	Standard for Zero Point	Component	Maximum Points	Standard for Maximum Point	Standard for Zero Point
Adequacy				Adequacy			
Total grains	5	≥2.5 SP/1000 kcal	No intake	Nuts and legumes		≥1 serving/d	No intake
Whole Grains and mixed beans	5	≥0.6 SP/1000 kcal	No intake	Whole grains	10	≥75 g/d	No intake
Tubers	5	≥0.3 SP/1000 kcal	No intake	PUFA	10	≥10% of energy	≤2% of energy
Total vegetables	5	≥1.9 SP/1000 kcal	No intake	Vegetables	10	≥5 servings/d	No intake
Dark vegetables	5	≥0.9 SP/1000 kcal	No intake	Long-chain (*n* − 3) fatty acids EPA + DHA	10	≥250 mg/d	No intake
Fruits	10	≥1.1 SP/1000 kcal	No intake	Fruit	10	≥4 servings/d	No intake
Dairy	5	≥0.5 SP/1000 kcal	No intake				
Soybeans	5	≥0.4 SP/1000 kcal	No intake				
Fish and Seafood	5	≥0.6 SP/1000 kcal	No intake				
Poultry	5	≥0.3 SP/1000 kcal	No intake				
Eggs	5	≥0.5 SP/1000 kcal	No intake				
Seeds and Nuts	5	≥0.4 SP/1000 kcal	No intake				
Moderation				Moderation			
Added sugars	5	≤10% of energy	≥20% of energy	Sugar-sweetened beverages and fruit juice	10	No intake	≥1 serving/d
Sodium	10	≤1000 mg/1000 kcal	≥3608 mg/1000 kcal	Sodium	10	≤1112 mg/d	≥3337 mg/d
Cooking oils	10	≤15.6 g/1000 kcal	≥32.6 g/1000 kcal	trans Fat	10	≤0.5% of energy	≥4% of energy
Red meat	5	≤0.4 SP/1000 kcal	≥3.5 SP/1000 kcal	Red/processed meat	10	≤1 serving/m	≥1.5 servings/d
Alcohol	5	≤15 g	≥40 g	Alcohol	10	0.5–1.5 drinks/d	≥2.5 drinks/d

**Table A2:** Linear regression analysis of the association between total CHEI score and socio-demographic, anthropometrics factors.

Variables	CHEI Score	Univariate Model		Multivariate Model	
	Mean (SD)	β (95%CI)	*p* Value	β (95%CI)	*p* Value
Age					
≤30	53.9 (7.6)	Reference		Reference	
>30	56.1 (8.0)	2.12 (0.59, 3.65)	0.007	0.77 (−1.22, 2.76)	0.028
Ethnicity					
Han ethnic	56.6 (8.4)	Reference		Reference	
Minority ethnic	53.1 (6.8)	−3.51(−5.03, −2.00)	0.000	−3.17 (−4.69, −1.65)	0.000
Education					
≤9 years	54.7 (7.3)	Reference			
10–12 years	55.4 (7.9)	0.76 (−1.15, 2.67)	0.43		
≥13 years	54.1 (8.2)	−0.57 (−2.94, 1.79)	0.65		
Occupation					
Unemployed	55.5 (7.6)	Reference			
Employed	54.5 (7.9)	−0.99 (−2.68, 0.70)	0.25		
Monthly family income ($)					
≤420	55.5 (8.2)	Reference			
>420	54.8 (7.8)	−0.69 (−2.47, 1.08)	0.443		
Monthly family income ($)					
≤420	54.7 (7.7)	Reference			
>420	55.2 (7.9)	0.497(−1.263, 2.258)	0.579		
Applied diet regulation for GDM control					
No	53.8 (7.8)	Reference		Reference	
Yes	55.9 (7.8)	2.08 (0.52, 3.63)	0.009	1.33 (−0.33, 2.99)	0.117
Applied physical activity for GDM control					
No	54.6 (7.7)	Reference		Reference	
Yes	56.6 (8.5)	1.99 (0.11, 3.88)	0.038	0.68 (−1.31, 2.67)	0.686
Children number					
1	53.9 (7.7)	Reference		Reference	
≥2	55.6 (7.9)	1.87 (0.28, 3.40)	0.021	0.645 (−1.01, 2.31)	0.465

Multivariate model *p* = 0.000.
